# Replication of Rocio virus in primary cultures of mouse neural cells

**DOI:** 10.31744/einstein_journal/2023AO0160

**Published:** 2023-05-23

**Authors:** Adriano da Paixão Souto, Samir Mansour Moraes Casseb, Karla Fabiane Lopes de Melo, Arnaldo Jorge Martins, Edna Cristina Santos Franco

**Affiliations:** 1 Universidade Federal do Pará Belém PA Brazil Universidade Federal do Pará , Belém , PA , Brazil .; 2 Instituto Evandro Chagas Belém PA Brazil Instituto Evandro Chagas , Belém , PA , Brazil .

**Keywords:** Rocio virus, Flavivirus, *Flavivirus* infection, Primary cell culture, Central nervous system infections, Antigens, viral, Disease models, animal, Mice

## Abstract

**Objective:**

This study verified the replication efficiency of the Rocio virus in a primary culture of mouse neural cells.

**Methods:**

Mixed primary cultures (neurons/glia) obtained from the brains of newborn isogenic BALB/c mice were inoculated with Rocio virus on the 7 ^th^ day of culture, and the development of cytopathogenic effects was monitored. The infection was confirmed via immunocytochemistry (anti-ROCV), while viral replication was quantified in infected primary cultures. The titration method used depended on the infection period.

**Results:**

Rocio virus efficiently infected primary cultured neural cells, with the highest viral titer causing cytopathic changes was observed at 2 days post infection. The virus-infected primary culture survived for up to 7 days post infection, and viral load quantitation showed viral replication kinetics compatible with the cell death kinetics of cultures.

**Conclusion:**

The findings of this study suggest that mouse neural cell primary cultures support Rocio virus replication and could be used as an alternative system for studying *Flavivirus* infection in the central nervous system.

**Figure:**
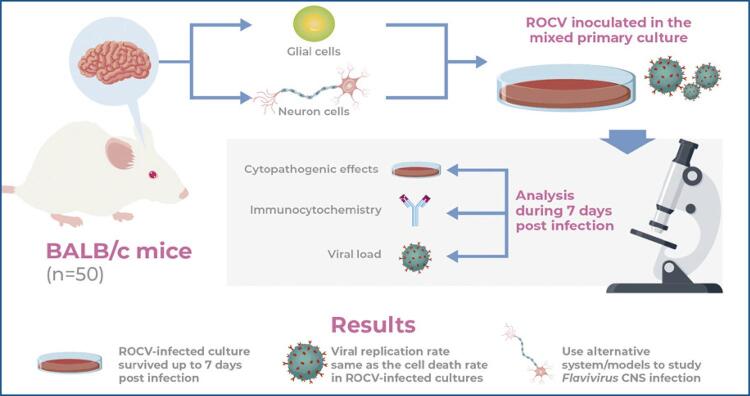

## INTRODUCTION

The Rocio virus (ROCV) is an arbovirus that causes encephalitis *.* It belongs to the family Flaviviridae and the genus *Flavivirus.* It was first isolated in 1975 in the southeastern region of Brazil from an outbreak of encephalitis in humans, affecting at least 23 countries in Ribeira Valley, São Paulo State. This outbreak began in 1973 and was curbed in 1980. The reason for this outbreak is still unclear. Approximately 1,021 encephalitis cases with a fatality rate of 10% were reported during the infection period. The ROCV disease is characterized by an acute onset of high fever, headache, prostration, myalgia, and vomiting. Neurological symptoms manifest as changes in consciousness, motor and reflex changes, balance disorders, mobility issues, and seizures. ^( 1 )^

The members of the genus *Flavivirus* are responsible for the significant morbidity and mortality associated with their infections. There are 36 Flaviviruses known to cause disorders in humans, and it is estimated that at least one of these viruses infects approximately two-thirds of the world’s population. Flaviviruses include the dengue virus, which infects approximately 50 million people each year; the West Nile virus (WNV), which has become endemic in the Northern Hemisphere since 1999 and continues to cause significant public health problems; the Yellow fever virus, which is endemic in African and South American countries; and the Japanese encephalitis virus (JEV), which is endemic in Asian countries and has a 30% to 40% mortality. ^( 2 )^

Considering the strong potential encephalitogenic nature of the ROCV, it is important to study the pathogenesis of this agent in systems that affect the central nervous system (CNS). However, the few studies conducted on ROCV so far have predominantly used *in vivo* experimental models, with no reports on the use of primary cultures of CNS cells as *in vitro* models for ROCV infection. This study was conducted to verify whether ROCV can replicate efficiently in primary cultures of mouse CNS cells. Our data demonstrate the cytopathogenic effect and death of primary CNS cultured cells due to ROCV replication. Additionally, the ROCV has active replication kinetics in this system five days post-infection. These findings open the possibility of developing an experimental infection protocol of mice primary neural cells of mice by ROCV, contributing greatly to future studies involving the replication of ROCV and other encephalitogenic *Flavivirus* and virus-neural cell interactions in vertebrates. ^( 1 )^

## OBJECTIVE

To verify the efficient replication of the Rocio virus in primary cultures of mouse neural cells.

## METHODS

### Viral stock

We used the Rocio virus strain (BeH34675, GenBank: MF461639) from a collection of viral isolates from the Section of Arbovirology and Hemorrhagic Fevers, *Instituto Evandro Chagas* (SAARB/IEC). For this study, a viral stock of ROCV strains was generated through VERO cell culture. Dulbecco’s Modified Eagle Medium (DMEM) (Gibco, USA) was used for the propagation and maintenance of cells. Cells were maintained at approximately 28°C, with weekly passages of confluent monolayers in 25cm ^2^ plastic bottles with 10mL growth medium. ^( 2 )^

The inoculated culture was observed daily under an inverted microscope to detect the cytopathogenic effect. When approximately 70% of the cells were infected, we used anti-ROCV polyclonal antibodies from SAARB/IEC and performed indirect immunofluorescence. The infected culture was maintained at -70°C. After 24 hours, the culture was defrosted and aliquoted into 1mL microtubes, which were stored at -70°C.

The viral titer, which indicates the number of infectious viral particles present in 1 mL of the viral stock, was determined by analyzing the culture plates and expressed as plaque-forming units per milliliter of culture medium (PFU/mL). The viral titer was calculated by applying the formula: PFU/mL = n x FC x 10d, where *n* is the average of the number of lysis plaques formed in the two wells inoculated with the highest dilution that generated isolated plaques; *FCc* is the correction factor, the number by which the value of the inoculum used must be multiplied to obtain 1mL (in this case, it was 10); and *10d* is the inverse of the dilution in which the value of *n* was found. ^( 3 )^

### Primary culture of neural cells

Male or female neonate isogenic mice of the BALB/c strain up to 2 days old from the IEC vivarium (n=50) were used. The animals used in the experiments were sacrificed via decapitation in compliance with the CONCEA Euthanasia Practice Guidelines. ^( 4 )^ Decapitation was performed outside the laminar flow cabinet in a Petri dish containing 70% ethanol. The animals’ heads were placed in a cabin for brain removal, which was performed on a Petri dish containing an ice-cold sterile dissection medium (Phosphate buffered saline). The brains were crushed with scissors and incubated for 5-10 minutes at 37°C in a solution of 0.05% calcium- and magnesium-free trypsin-EDTA so that the tissue could undergo enzymatic digestion. Later, the material was transferred to a 15mL falcon tube containing DMEM with 10% fetal bovine serum (FBS) and mechanically crushed with a Pasteur pipette. The cell suspension was left to rest for 2 minutes so that excess brain material could be decanted and the supernatant be collected. The cell density of the supernatant was measured using a Neubauer chamber with 50µL of Trypan blue, 40µL of saline, and 10µL of the cell suspension.

To obtain mixed cultures (neurons/glia), the cells in the supernatant were grown at a density of 5×10 ^5^ cells/well in 24-well culture plates (15.4mm in diameter and 1.91cm ^2^ per growth area) whose wells were covered with poly-L-lysine. Each well also contained 1.5mL of neurobasal culture medium supplemented with 10% FBS, 25µM glutamate, 100IU/mL penicillin, 0.1mg/mL streptomycin, 1mM GlutaMAX ^TM^ (Gibco), and 0.25mM L-glutamine. The plates were kept in an incubator at 37 °C in a humidified atmosphere with 5% carbon dioxide (CO ^2^ ). After 24 hours, this medium was changed, and the spent medium was changed every three days thereafter. ^( 5 )^

### Inoculation of ROCV in primary cultures of neural cells

The volume of the viral suspension of ROCV from the viral stock used as the inoculum had a multiplicity of infection index (MOI) of 2. This MOI was selected based on the previous work of Henriques et al. ^( 2 )^ The culture medium was completely removed, and the inoculum containing approximately 1×10 ^6^ infectious virions was inoculated. The treated wells, as negative controls, were inoculated using sterile neurobasal culture medium only, and the cells were subjected to the same conditions as those inoculated with ROCV. The cultures were maintained at 37 °C in an incubator with 5% CO _2_ in a humidified atmosphere and observed for cytopathic changes daily using an inverted microscope.

### Phenotypic characterization of the primary neural cell cultures and detection of ROCV viral antigen via immunocytochemistry

Immunocytochemical tests were carried out using an immunoassay kit (DAKO, EUA) according to the manufacturer’s protocol. For phenotypic characterization of primary cultures of neural cells, neurons were labeled with antibodies against the NeuN protein (anti-NeuN) (DAKO, EUA), astrocytes were labeled with antibodies against glial fibrillary acidic protein (anti-GFAP) (Thermo Scientific, EUA), and microglia were labeled with antibodies against adapter-ionized (AIP) calcium-binding molecule 1 (anti-IBA 1) (DAKO). The immunohistochemical pattern of cultures was obtained by calculating the percentage of labeled cells (NeuN, GFAP, and IBA 1) in relation to the total number of cells stained, as previously reported by Kádár et al. ^( 6 )^

The cells were analyzed using an inverted microscope. Control wells not treated with the primary antibodies were included in the test.

## RESULTS

### Viral stock characterization

**Figure 1:**
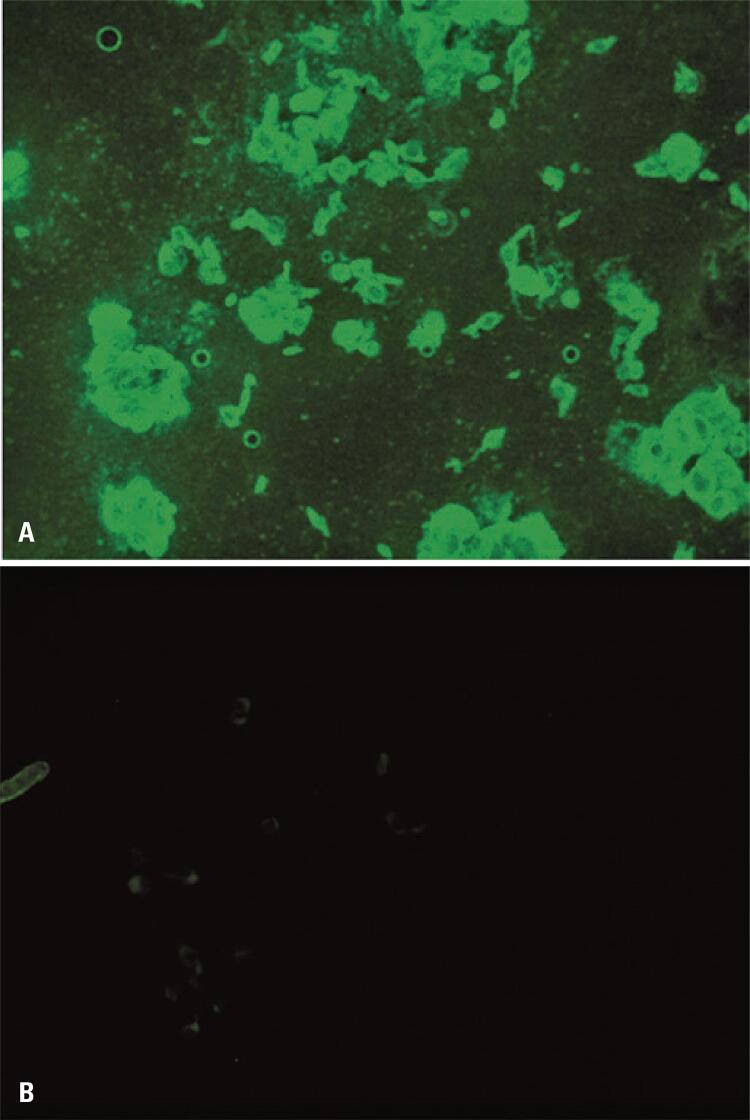
(A) Indirect immunofluorescence of VERO cells infected with Rocio virus at 4 days post infection as labeled using polyclonal anti-ROCV antibodies; (B) Indirect immunofluorescence of uninfected (control) VERO cells. Magnification: 200×

The culture of VERO cells inoculated with the viral suspension prepared from the brains of sick animals showed cytopathogenic effects from 2 days post infection; approximately 70% of the cells showed cytopathogenic effects at 4 days post infection. The immunofluorescence test performed on samples from this culture showed strong reactivity (+++) for the ROCV antigen ( Figure 1A ) compared with the negative control ( Figure 1B ). The viral stock had a titer of 4.7×10 ^6^ PFU/mL.

### Phenotypic characterization of primary neural cell cultures

**Figure 2:**
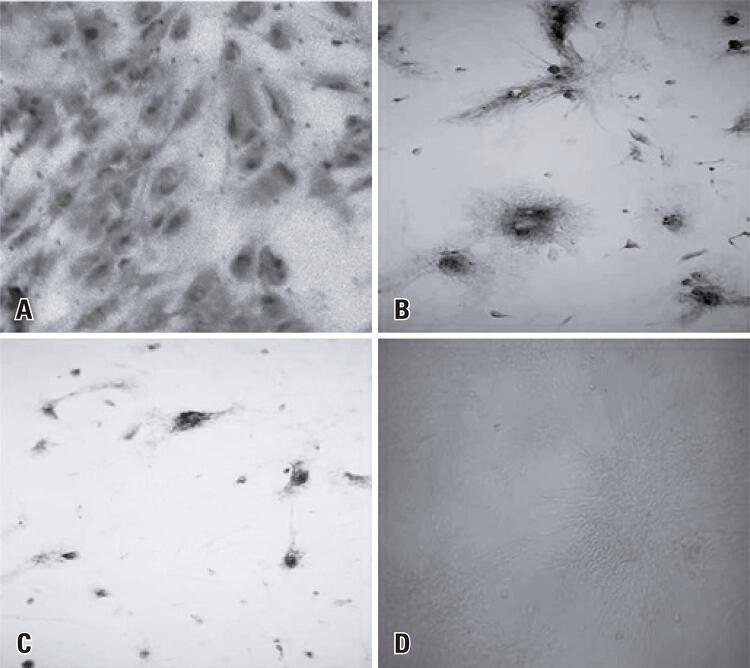
Immunocytochemistry of the primary mouse neural cell cultures. (A) Staining with anti-NeuN antibodies; (B) Staining with anti-GFAP antibodies; (C) Labeling with anti-Iba-1 antibodies; (D) Control cells incubated without primary antibodies. Magnification: 200×

Identification of the cell phenotype present in primary neural cell cultures via immunocytochemistry experiments was performed to detect neurons (anti-NeuN), astrocytes (anti-GFAP), and microglia (anti-IBA 1). The results revealed a heterogeneous cell composition of these three cells ( Figure 2 ). These experiments were repeated four times to ensure data reliability and reproducibility. Counterstaining using the Nissl method revealed that primary cultures consisted of 39% neurons, 28% astrocytes, and 23% microglia.

The Nissl method uses cresyl violet staining for free-floating sections, which are then mounted and air-dried. A cresyl violet acetate solution was used to stain the cytoplasm of neurons in paraformaldehyde- or formalin-fixed tissues, and the neutrophils was stained with purple-blue granules.

### Kinetics of death of primary neural cell cultures infected with the ROCV

**Figure 3:**
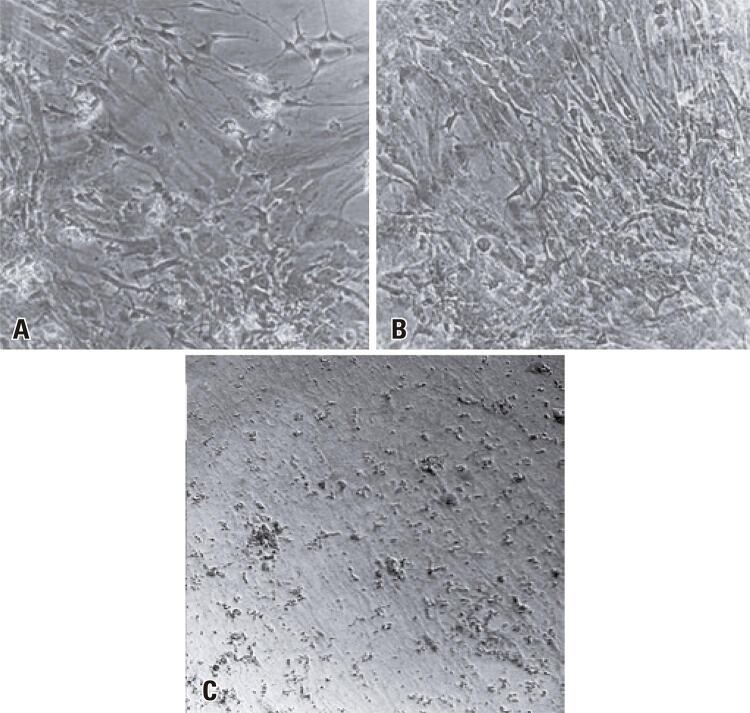
Primary culture of mouse neural cells infected with the Rocio virus. (A) A monolayer of uninfected neural cells at 5 days post infection cultured with sterile neurobasal culture medium; (B) Rocio virus-infected neural cells at 2 days post infection; (C) Rocio virus-infected neural cells at 7 days post infection. Magnification: 200×

Primary cultures of neural cells were inoculated with ROCV on the 7 ^th^ day of culture when the monolayer of neural cells was established and appeared sufficiently dense. After inoculation, the negative control remained healthy for up to six days as no morphological changes were observed during this period ( Figure 3A ); four replicates were conducted to assure data dependability. From the 7 ^th^ days post infection, the culture began to die, with cells disintegrate and progressively dying at the 10 ^th^ days post infection. Compared to the negative control cells, the cells inoculated with ROCV began to show cytopathogenic effects from the 2 ^nd^ days post infection ( Figure 3B ), with few cells exhibiting morphological changes, such as granules and cell aggregation, and a slight regression of the cell monolayer, which remained until 5 ^th^ days post infection. From the 6 ^th^ days post infection ( Figure 3C ).

**Figure 4:**
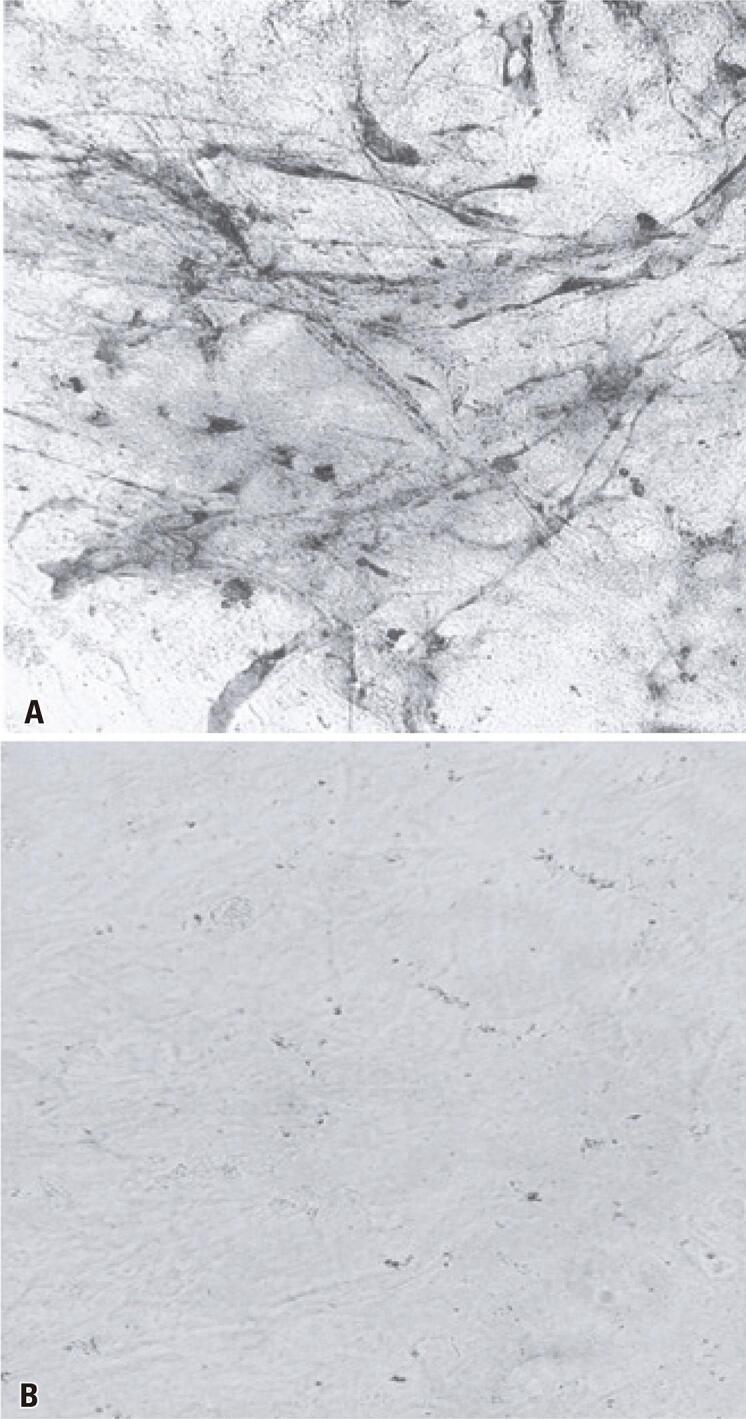
(A) Immunocytochemistry of primary mouse neural cell cultures infected with Rocio virus at the 5 th days post infection and incubated with polyclonal anti-ROCV antibodies; (B) Immunocytochemistry of uninfected primary mouse neural cell cultures and incubated with polyclonal anti-ROCV antibodies. Magnification: 200×

ROCV antigens were detected in the primary neural cell cultures via immunocytochemistry. At 5 days post infection, the cells revealed strong reactivity (+++) ( Figure 4A ) compared to the negative control, which was inoculated with sterile neurobasal culture medium only ( Figure 4B ). Four replicates of these experiments were conducted to ensure data dependability.

### ROCV replication in primary neural cell cultures

Quantifying viral replication during the infectious period was performed via virus titration using samples of the spent primary culture medium following infection with ROCV at 5 days post infection. The virus titers observed at 1, 2, 3, 4, and 5 days post infection were 1×10 ^7^ , 1×10 ^8^ , 1×10 ^7^ , 1×10 ^6^ , and 7×10 ^5^ PFU/mL, respectively.

## DISCUSSION

Primary cultures of neural cells are paramount to studying CNS *Flavivirus* infections, as they allow us to explore the intrinsic behavior of CNS cells without the interference of blood components. Several protocols for obtaining primary cultures of CNS cells have been published, encompassing various compositions of neurons and glial cells and their mode of action. ^( 5 , 7 , 8 )^

In this study, we have optimized the primary cultures of neural cells to create a representative system of the CNS, *i.e.,* containing neurons and glia. The cell monolayers were sufficiently dense and established from the 7 ^th^ day of cultivation. Immunocytochemistry and counterstaining using the Nissl method revealed a heterogeneous cell composition, with the three CNS cell types present: neurons, astrocytes, and microglia ( Figure 2 ). These results corroborate the results obtained by Gao et al., ^( 5 )^ Ju et al., ^( 7 )^ and Zhang et al., ^( 8 )^ validating the paradigm of this study as an *in vitro* experimental model for the study of CNS infections.

Based on the cell death profile, *i.e* viral antigen detection by immunocytochemistry, viral titer in infected cells; enable determination of efficient replication of ROCV in neural cells. To create favorable conditions for both viral replication and the growth of primary neural cell cultures, the cells were inoculated with ROCV on the 7 ^th^ day of astrocytes, causing dense monolayer growth of neural cells.

Considering the neurotropism of ROCV, a broad permissiveness in the primary cultures was expected in this study after ROCV infection. This causes rapid cell destruction leading to complete death of the culture up to a maximum of 4 ^th^ days post infection, as shown by Diniz et al. ^( 9 )^ in a model of neuronal infection using WNV. Interestingly, the cultures inoculated with ROCV showed cytopathogenic effects and subtle cell death, observed only from the 2 ^nd^ days post infection ( Figure 3B ), which remained constant until the 5 ^th^ days post infection. From 6 days post infection, cell death increased, and the number of cells in the cell monolayer until the complete death of the culture on the 7 ^th^ days post infection ( Figure 3C ).

This profile of primary neural cell culture death is similar to the findings of primary glial cell culture death after infection with JEV as obtained by Chen et al. ^( 10 )^ In addition, Kumar et al. reported that the encephalitogenic *Flavivirus* generated similar profiles of cell death in cultured human neuroblastoma cells. ^( 11 )^ The negative control cultures remained healthy for up to six days, then began to show signs of cell death and gradual disintegration of the cell monolayer from 7 ^th^ days post infection along with complete death of the culture on the 10 ^th^ days post infection. The death of the negative control cultures resulted from nutrient depletion in the growth medium. Since the control cells were not introduced to fresh media, the same conditions as ROCV-infected cultures were maintained.

Aspects inherent to the particular physiology of CNS cells should also be considered in the kinetics of primary culture death. It has been proposed that viral infections can induce the death of infected cells via necrosis or apoptosis due to inflammatory responses. ^( 12 - 14 )^ Apoptosis of cells infected by *Flavivirus* can occur either directly as a result of viral spread or indirectly via mobile sensors that initiate cell death. Thus, apoptotic death serves as a mechanism of the innate immune response. ^( 14 , 15 )^ However, in CNS infections, neural cells regulate their immune responses to preserve the integrity of nerve tissue as they try to eliminate the virus since severe neuronal damage can cause functional damage in the host. ^( 16 - 18 )^

Thus, neurons and many glial cells are highly resistant to the induction of apoptosis, which is mediated by viral replication or cytotoxic cells. This is likely because of the expression of apoptotic inhibitors, such as Bcl-2, Bcl-xL, and Mcl-1, which can be activated as nerve tissue protection mechanisms. ^( 19 , 20 )^ Additionally, neurons regulate the expression of interferons, which are responsible for the antiviral response activation in adjacent cells. ^( 16 )^ It has also been proposed that astrocytes contribute to the survival of CNS mixed primary cultures because they are more resistant to viral infection and regulate the spread of WNV in the CNS, possibly causing persistent infection. ^( 9 , 17 , 18 )^

To quantitatively determine viral replication in primary ROCV-infected cell cultures depending on the infectious period, viral load was quantified using the viral titration method at intervals of 24 hours during the first five days of the infectious period. We chose this period by considering the profile of observed cell death in primary cultures inoculated with ROCV. Interestingly, the ROCV replication kinetics results we obtained were similar to the WNV replication kinetics in neural cell cultures obtained by Kumar et al. ^( 11 )^ and van Marle et al. ^( 19 )^

## CONCLUSION

In conclusion, the current study reports that primary cultures of neural cells from newborn mice enable Rocio virus infection. The infection causes a mild cytopathogenic effect on the cells at 2 days post infection. The highest viral titer was observed at 5 days post infection, and culture death gradually began at 7 days post infection. Our results demonstrate the advantage of culturing primary neural cells and infecting them with the Rocio virus as an alternative central nervous system infection system to replace the *in vivo* model, which is practical, faster, and affordable. Moreover, this system provides an elaborate analysis of the infectious process at the cellular level and contributes to the immunologic and pathophysiological exploration of Rocio virus infection and encephalitogenic Flaviviruses.
